# Retraction: Predictors of Quality of Life in Patients With Rheumatoid Arthritis in Pakistan: A Cross-Sectional Study

**DOI:** 10.7759/cureus.r42

**Published:** 2022-03-17

**Authors:** Rejja Irfan, Sohaib Tousif, Romaisa R Khan, Asma Bham, Khizer Shamim, Rahil Barkat

**Affiliations:** 1 Internal Medicine, Brooklyn Cancer Care, New York, USA; 2 Medicine, Ziauddin Medical University, Karachi, PAK; 3 Internal Medicine, Liaquat National Hospital and Medical College, Karachi, PAK; 4 Indus Hospital Research Center, Indus Hospital & Health Network, Karachi, PAK; 5 Internal Medicine, Ziauddin University Hospital, Karachi, PAK; 6 Indus Hospital Research Center, The Indus Hospital, Karachi, PAK

This article has been retracted and the contents removed due to data theft and purchased authorships. It was discovered that Rahil Barkat (last author) used research data without the knowledge or permission of the principal investigator, a colleague of Mr. Barkat, who was not listed as an author.

Upon discovering the published article during a routine literature review, the principal investigator notified her institution, Indus Hospital & Health Network, which conducted a thorough investigation confirming the data theft as well as the inclusion of several co-authors who were asked by Mr. Barkat to proofread the article and provide payment in exchange for authorship. These payments were made in the guise of “editing fees” but greatly exceed any editing fees paid to Cureus. While these authors may have been defrauded by Mr. Barkat, they remain complicit due to their lack of honest contributions to the article. (Proofreading is an insufficient contribution to warrant authorship as defined by ICMJE.) The article contents have been removed to allow for the rightful owners of this data to publish it at their discretion.

Upon receiving the results of this investigation, Cureus immediately undertook a comprehensive review of all other Cureus publications from Mr. Barkat and his frequent co-authors, which revealed a consistent pattern of fraud and resulted in the retraction of all articles.

